# Environmental DNA Detection of the Golden Tree Frog (*Phytotriades auratus*) in Bromeliads

**DOI:** 10.1371/journal.pone.0168787

**Published:** 2017-01-04

**Authors:** Jack D. Torresdal, Aidan D. Farrell, Caren S. Goldberg

**Affiliations:** 1 Independent Scholar, Portland, Oregon, United States of America; 2 Department of Life Sciences, The University of the West Indies, St. Augustine, Trinidad and Tobago; 3 School of the Environment, Washington State University, Pullman, Washington, United States of America; University of Innsbruck, AUSTRIA

## Abstract

The analysis of environmental DNA (eDNA) is a powerful, non-destructive technique for detecting rare or hard to find freshwater organisms. In this study, we investigated the effectiveness of environmental DNA analysis as a method for detecting a rare amphibian, the golden tree frog (*Phytotriades auratus*). These frogs are believed to live exclusively within one species of tank bromeliad, *Glomeropitcairnia erectiflora*, found on the highest peaks of the island of Trinidad in the West Indies. Previous survey methods for this species involved bromeliad destruction, while here we collected and analyzed water samples from discrete pools within *G*. *erectiflora* plants for species-specific DNA. We found 1) that we can identify the presence of *P*. *auratus* in the bromeliads using environmental DNA analysis, and 2) that environmental DNA evidence indicates the presence of a previously undiscovered *P*. *auratus* population, increasing the species’ range from two isolated ‘sky islands’ to three.

## Introduction

The use of environmental DNA (eDNA) as a method for detecting rare and elusive species has been successfully implemented in a range of freshwater systems, including wetlands, streams and lakes [1,2]. Environmental DNA describes genetic material that has been released into the environment, and the detection of macro-organisms using this material has enormous potential to contribute to conservation through detection of previously unknown populations of rare species. One such species is the golden tree frog (*Phytotriades auratus*) which completes most of its life cycle within the tank of an epiphytic bromeliad [3, 4]. Surveys for this species are challenging due to their small size (3–4 cm) and complex habitat; they spend the majority of their lives hidden within the leaf whorls of the epiphytic giant tank bromeliad *Glomeropitcairnia erectiflora*. The frog is reclusive, and is seldom seen out of the pools of water caught in the bromeliads, where it is thought to breed throughout the year [4]. The International Union for Conservation of Nature (IUCN) lists *P*. *auratus* as Critically Endangered because of its small range [4], which is reported to be approximately 206 ha on two mountain peaks on the island of Trinidad [3]. Additionally, the distribution of this species is severely fragmented, and there is continuing decline in the extent and quality of its habitat, the Elfin Woodland [5]. Records are scarce for *P*. *auratus* and previous surveys have been unsuccessful at confirming its range, density, or population sizes [3]. The species is thought to be highly vulnerable to climate change due to its distribution only on high mountain peaks, thus offering no potential for a range shift to a higher altitude as climates warm [6].

Traditional field sampling for *P*. *auratus* involves removing or destroying the host plant, and such sampling is considered one of the main threats to the survival of this species [3]. Recently, the Trinidad and Tobago Environmental Management Act of 2013 (Legal Notice No. 32), which aims to protect environmentally sensitive species, specifically prohibited the “collection, destruction or removal of *G*. *erectiflora*.” As such, there is a pressing need for an alternative method for detecting and monitoring populations of *P*. *auratus*. As eDNA collection involves collecting water samples non-destructively, it is consistent with habitat protection. Additionally, eDNA sampling with single-use instruments minimizes the risk of spreading the chytrid fungus, *Batrachochytrium dendrobatidis* (Bd), which exists in Trinidad [7] and has been found in bromeliad water elsewhere [8]. In this paper, we demonstrate that eDNA from bromeliad water samples is an effective method for detecting *P*. *auratus* in these sensitive environments and that it can be used to detect evidence of previously unrecorded populations.

## Materials and Methods

### Site selection

**Fig 1:**
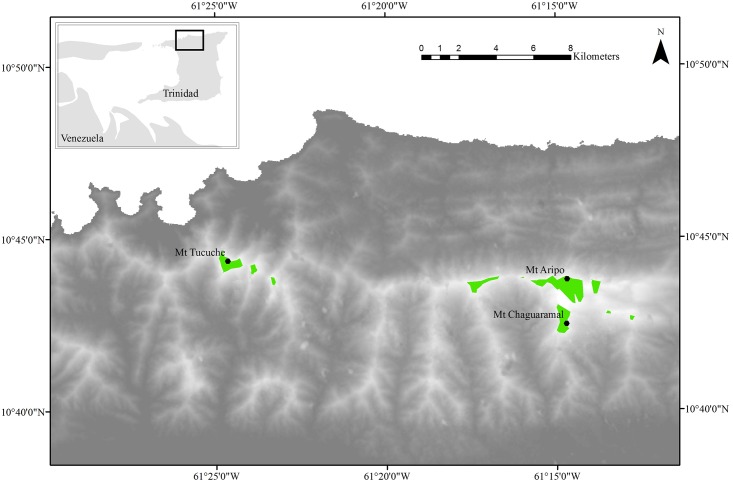
Map of Trinidad’s northern range showing sampling sites and elevation (30 m resolution). Green polygons indicate where elevations are above 750 m.

We sampled bromeliads on three mountain peaks in the Northern Range of Trinidad, West Indies. An elevation map was derived to show the collection locations (Fig 1). Data for the digital elevation model was extracted from the publicly available 30 m resolution ASTER GDEM database [9], using ArcMap Geographical Information System software (ArcMap 10.2.1; Esri, Redlands, California). Two of these peaks, El Tucuche and El Cerro del Aripo, are known to have populations of *P*. *auratus*. The species has not been recorded on the third peak, Chaguaramal, despite *G*. *erectiflora* having been reported on this peak [3]. We individually sampled 28 bromeliads from a total of six trees (three on El Tucuche, two on El Cerro del Aripo, and one tree on Chaguaramal), and one bromeliad on the forest floor of El Tucuche. The height of the bromeliads ranged from 3–8 m and were accessed using tree climbing equipment.

#### Sample collection

We collected water samples from the bromeliads using single use 100 ml syringes. We used new gloves and a new syringe for each sample to avoid cross contamination. Each volume collected was stored in a sterile whirlpak until filtered. Sample volumes varied based on the size of the bromeliad. We filtered all samples on site after descending from the tree canopy alongside one sample negative (100 ml molecular water) per site. We concentrated eDNA from the water samples using 0.45 μm cellulose nitrate filter funnels (Nalgene, Inc.) as in Goldberg et al. [10].

We also collected DNA samples from any frog species seen or known to be seen on or near these peaks for eDNA assay validation using mouthswabs. Non-target species consisted of *Pristimantis urichi*, *Flectonotus fitzgeraldi*, *Mannophryne trinitatis* and *Scinax ruber*. In addition, we received two archived *P*. *auratus* tissue samples (UWIZM.2012.27.73, UWIZM.2010.9.28) from the University of the West Indies Zoology Museum, which is a publicly accessible collection and a permanent repository. Mouthswabs were collected in compliance with “Guidelines for use of live amphibians and reptiles in field and lab research,” from the Herpetological Animal Care and Use Committee of the American Society of Ichthyologists and Herpetologists, 2004. All collections were made under permits from The Wildlife Section of the Forestry Division, Ministry of the Environment and Water Resources, Trinidad & Tobago.

### Laboratory analysis

We extracted all water filter samples in a lab dedicated to low quality/low quantity DNA samples at Washington State University. This laboratory had restricted access; researchers were required to shower and change into fresh clothing before entering after having been in the tissue laboratory or any room where PCR products were created or handled. Filter extractions were conducted using the Qiashredder/DNeasy protocol [10]. Tissue extractions were conducted using the DNeasy Blood & Tissue Kit (Qiagen, Inc.) in a separate lab. Negative extraction controls were created with each batch of extractions.

We designed a species-specific quantitative PCR (qPCR) assay for *P*. *auratus* from cytochrome b sequences stored in Genbank (NCBI; [11]) using Primer Express (Applied Biosystems), targeting an 80 bp fragment. We screened potential designs for cross-species amplification first using PrimerBlast (NCBI) with settings of at least 2 base pair changes on each primer with at least 1 in the last 4 base pairs from the 3’ end. We confirmed a design that would not cross-amplify with any other species on the island (Table 1), given the sequences in the database. For the next stage of validation, we tested this design against extracts of tissue samples from one sample of the target species (the other sample did not yield DNA, potentially due to formalin preservation) and at least one tissue sample of all additional amphibians known to exist on these peaks. We conducted all qPCR analyses in 15 μl reactions using QuantiTect Multiplex PCR Mix (Qiagen, Inc.) with recommended multiplexing concentrations (1X QuantiTect Multiplex PCR mix and 0.4 μM of probe and each primer) on a CFX96 Touch^™^ Real-Time PCR Detection System (BioRad). Cycling began with 15 min at 95°C followed by 50 cycles of 94°C for 60 s and 60°C for 60 s. After validation, we used this assay to detect eDNA of *P*. *auratus* in the collected filter samples. We included an exogenous internal positive control (Applied Biosystems) in each well to test for sample inhibition. All samples were analyzed in triplicate, with an additional triplicate reaction for any sample that tested positive only once in the original reaction. Plates were analyzed with a gBlock gene standard (Integrated DNA Technologies) with a standard curve consisting of 10-fold dilutions of 1000–1 copies/well in duplicate. Quantitative results were obtained by averaging the quantities of the first three replicates for all samples testing positive, including zero values. We tested the limit of detection of this assay by analyzing 10 wells each of 1, 3, 5, and 10 copies of the standard. For new locations, the product of the *P*. *auratus* assay was sequenced by the University of Arizona Genetics Core.

**Table 1:** Species-specific quantitative PCR assay for *Phytotriades auratus*.

Forward primer	GCGGATTCTCTGTCGACAATG
Reverse primer	TGCTCCTGCAATAAGAAATGGA
Probe	6FAM-CTCTCACCCGATTTTTTACAT-MGB

### Statistical analysis

We tested whether amount of eDNA in a sample (log transformed) when the species was detected was related to volume sampled using linear regression and bromeliad size with an ANOVA using R version 3.3.1 [12].

## Results

The assay for *P*. *auratus* passed all stages of validation, with 5 copies being the lowest amount where all 10 wells tested positive. All eDNA and extraction negative controls tested negative in the assay and inhibition was not detected in the eDNA samples. Sighting of frog species in the field provided further verification of the method. During sampling we recorded one confirmed *P*. *auratus* in a single bromeliad on el Cerro del Aripo. We collected two eDNA samples of different volumes from this bromeliad (Table 2); both samples (A11, A12) tested positive. The samples where a different frog species was present in the bromeliad (A06 and ET10; *F*. *fitzgeraldi*) tested negative, again indicating a lack of cross-species amplification (i.e. false positives).

**Table 2:** Environmental DNA sample results from bromeliads in three mountain ranges across Trinidad, West Indies. Results are ordered by tree and by total bromeliad diameter in meters, small (S < 0.33 m), medium (M 0.33–0.66 m), or large (L > 0.66 m). Total eDNA copies is the copy number estimated for the whole sample.

Mountain Peak	Tree ID	Sample ID	Volume (ml)	Bromeliad Size	Frog Visual	Positive replicates	Total eDNA copies
Cerro del Aripo	1	A11	150	L	*P*. *auratus*	3/3	357878
	1	A12	50	L	*P*. *auratus*	3/3	54459
	1	A08	75	L		2/3	45
	1	A09	100	L	Tadpole	3/3	56984
	1	A10	100	L		3/3	579
	1	A06	50	M	*F*. *fitzgeraldi*	0/3	0
	1	A07	50	M		3/3	503878
	2	A01	100	M		3/3	948
	2	A02	100	M		0/3	0
	2	A03	100	M		0/3	0
	2	A04	100	M		3/3	3244
	2	A05	100	M		3/3	6833
El Tucuche	1	ET11	150	L		3/3	255
	1	ET08	235	L		0/3	0
	1	ET06	150	M		3/3	288
	1	ET07	30	S		2/3	35
	1	ET09	120	S		3/3	597
	1	ET10	150	S	*F*. *fitzgeraldi*	0/3	0
	1	ET12	175	S		3/3	185
	2	ET03	175	L		3/3	211615
	2	ET02	125	M		3/3	4069
	2	ET01	125	M		3/3	177
	2	ET04	30	S		2/6	23
	3	ET05	100	M		3/3	10972
	Ground	ET13	100	*M*		2/6	152
Chaguaramal	1	CHA03	200	L		3/3	168
	1	CHA04	250	L		0/3	0
	1	CHA01	150	M		3/3	1562
	1	CHA02	100	M	Unknown Frog	3/6	23

Overall, of the 29 eDNA samples, 23 tested positive for *P*. *auratus* DNA (Table 2). The positive samples included a bromeliad where an unidentified frog was seen and a bromeliad where an unidentified tadpole was seen. There was no association between sample volume and eDNA copies captured (F_1,21_ = 0.13, p = 0.72); even the smallest sampled volumes used (30 ml) were sufficient to detect *P*. *auratus* eDNA. Similarly, there was no clear association between bromeliad size and the sample results (F_2,20_ = 2.39, P = 0.12; Table 2), even when standardized for volume sampled (F_2,20_ = 0.015, P = 0.98). Products from the three bromeliads testing positive on Chaguaramal had sequences that confirmed identification as DNA from *P*. *auratus*.

## Discussion

Conservation of a rare species must begin with knowledge of their distribution. In this study, we demonstrated a non-destructive method that was successful for detecting a rare amphibian that was previously impossible to detect without destroying its habitat. If, as is thought, the entire life cycle of *P*. *auratus* takes place in the bromeliad pools, there is a high likelihood of detecting the species in a forest using this method. We also used the method to detect, for the first time, the presence of *P*. *auratus* DNA on Chaguaramal, expanding the likely species range within Trinidad to three peaks. In the future, this method can be used to determine whether the frog's distribution is solely determined by the presence of its host plant, *G*. *erectiflora*, or if other ecological factors, such as temperature, moisture, and elevation are also critical.

Although the eDNA detection method used has clear advantages, care must be taken in interpreting the results. Presence data cannot be directly used to estimate the density of the species within a habitat patch because we do not know how often individuals move between bromeliads within a tree or between trees and how many individuals may occupy a single bromeliad. Additionally, small amounts of eDNA may be moved within habitat patches by other animals, such as birds. Nonetheless, the occurrence of several negative samples (approximately 20%), suggests that the results could be informative at a bromeliad scale.

Historically, *P*. *auratus* was only known on the second highest peak in Trinidad, El Tucuche. In the 1980s, Morley Read discovered the frog on El Cerro del Aripo [13] and later studies have confirmed its presence on both peaks [3,11]. There have also been recent sightings of individual frogs from the summit of Cerro Humo in Eastern Venezuela [14]. These reports, plus our discovery of the frog on Chaguaramal, leads us to believe that further undocumented populations may exist. *Glomeropitcairnia*. *erectiflora* populations have been reported on all four of Trinidad’s highest peaks (Cerro del Aripo, El Tucuche, Chaguaramal and Morne Bleu), although not consistently; *G*. *erectiflora* was reported on three of the four peaks in 1946, four of four in 1969, two of four in 1995 and two of three in 2008 [3,11,15,16]. We have observed *G*. *erectiflora* on all four peaks; based on the known distribution of *G*. *erectiflora* within Trinidad, we estimate that the maximum potential range extent for *G*. *erectiflora* in Trinidad is 415 ha. This includes all land above 750 m, which is the lowest elevation at which *G*. *erectiflora* has been recorded (Fig 1). This area consists of nine distinct sky islands, ranging in size from 3–182 ha. In most cases the peaks are close together, but others comprise more isolated patches of possible habitat. All of these areas are currently under forest cover, although in some cases the original forest cover has been disturbed by human activity [17]. The proposed area of 415 ha is approximately twice as large as that estimated by Clarke (1995)[3], which presented a predicted distribution limited to the two highest peaks (having failed to find *G*. *erectiflora* elsewhere). It should be noted that for peaks where the presence of *G*. *erectiflora* has been confirmed, dense populations have only been recorded at >800 m in association with Elfin Woodland, therefore it is unlikely that *P*. *auratus* will occupy all of the areas in Fig 1, assuming there is a threshold density for *P*. *auratus* habitat. Nonetheless, given the scarcity of both species we recommend that future work considers all of these areas for study and/or protection. We envisage that the method described here will provide a valuable tool in this effort.

Environmental sampling for DNA is particularly useful in this scenario, where field surveys cannot be conducted without habitat destruction or degradation using current sampling methods. Environmental DNA sampling from bromeliads has many potential applications across the world, yet examples in the literature are few, and deal primarily with microorganisms and antibiotic culturing [8, 18]. Environmental DNA could further be used to determine community structure patterns of bromeliad-dwelling species, including other amphibians and insects. In Trinidad, this is of particular interest on El Cerro del Aripo, where *F*. *fitzgeraldi* uses two tank bromeliad species, *G*. *erectiflora* and *Vriesia glutinosa*, and where *P*. *auratus* uses only *G*. *erectiflora*. Outside of Trinidad, application of this method could extend to arboreal, bromeliad-dwelling mesoamerican salamanders, whose populations exist in small or isolated patches of habitat [19, 20, 21]. Additionally, large tank bromeliads can harbor a rich fauna of invertebrates, including insects, myriapods, oligochaetes, arachnids and crustaceans [3]. Species interactions and habitat partitioning by these bromeliad dwelling species are critical to the functioning of these ecosystems. Here we have demonstrated the first steps towards disentangling the interactions inherent in these complex and understudied communities.
